# A qualitative study on traditional healers’ perceptions and management of epidermolysis bullosa

**DOI:** 10.4102/hsag.v28i0.2266

**Published:** 2023-08-28

**Authors:** Antoinette V. Chateau, Nceba Gqaleni, Colleen Aldous, Ncoza Dlova, David Blackbeard

**Affiliations:** 1 Department of Dermatology, Grey’s Hospital, Pietermaritzburg, South Africa; 2 Department of Dermatology, Faculty of Health Sciences, University of KwaZulu-Natal, Durban, South Africa; 3 Discipline of Traditional Medicine, School of Nursing and Public Health, University of KwaZulu-Natal, Durban, South Africa; 4 Africa Health Research Institute, Durban, South Africa; 5 Department of Genetics, Faculty of Health Sciences, University of KwaZulu-Natal, Durban, South Africa; 6 Department of Clinical Psychology, Grey’s Hospital, Pietermaritzburg, South Africa; 7 Department of Psychiatry, Faculty of Health Science, University of KwaZulu-Natal, Durban, South Africa

**Keywords:** traditional health practitioners (THPs), epidermolysis bullosa (EB), traditional practices, rare skin disease, South Africa

## Abstract

**Background:**

Epidermolysis bullosa (EB) is a rare, incurable genodermatosis causing blisters that can result in multisystemic complications and death. Limited data exists on EB in South Africa. Research indicates that the majority of African patients consult traditional health practitioners (THPs) before seeking allopathic healthcare.

**Aim:**

This study aims to understand THPs belief systems, experiences, perceptions and management of EB patients and their families in the social and cultural context to improve the healthcare of EB patients.

**Setting:**

The study setting is Nelson Mandela School of Medicine, Durban, and Grey’s hospital, Pietermaritzburg, KwaZulu-Natal.

**Methods:**

Qualitative in-depth interviews were conducted with 10 THPs. A non-probability, purposive sampling method was used. A two-site qualitative study was guided by interpretative phenomenological analysis. Guba’s trustworthiness framework was used to ensure rigour.

**Results:**

Three male and seven female THPs were interviewed, including sangoma, inyanga and umthandazi. The integration presented five global themes: (1) THP practices, (2) perceptions of THP, (3) experiences of THP with patients with EB, (4) diagnosis and management plans of THP and (5) vision and role of THPs. There were multiple divergent perspectives among the THPs with the shared African worldview.

**Conclusion:**

Understanding THPs belief systems and therapeutic options is crucial for holistic patient management. Knowledge exchange can promote safe healthcare practices and facilitate collaboration between traditional and allopathic health practitioners.

**Contribution:**

This is the first study to explore THPs perceptions and practices regarding EB, a rare disease.

## Introduction

Epidermolysis bullosa (EB) is a rare inherited bullous dermatosis that presents with mucocutaneous fragility (Has et al. 2020). Complications include pain, infections, skin cancer, gastrointestinal, skeletal, lung, heart and kidney pathology (Has et al. 2020). There is no cure for this disorder, and management aims to prevent blister formation and symptomatic care. Thereare very few studies on this rare skin disease in South Africa (SA), and none seek to understand the care practices and role of the traditional health practitioner (THP) in managing these patients.

A great demand is placed on the healthcare sector because of both communicable and non-communicable diseases. A further burden is placed by the influx of patients from surrounding countries who cross the border in search of medical assistance (Mokgobi 2014).

Sixty per cent of people in sub-Saharan Africa live in rural areas where Western medicine is not easily accessible or available, and traditional medicine is widely used to prevent and treat illnesses (Mutola, Pemunta & Ngo 2021). A total of 60% – 70% of the rural South African population seek the help of a THP before consulting a medical doctor; many continue to see a THP after consulting with a medical doctor (Mokgobi 2013; Shizha & Charema 2012). In 2010, Ross et al. documented approximately 250 000–400 000 THPs in SA compared to the 28 000 allopathic healthcare providers (Ross 2010). In 2019, Makaula et al. noted that there were 265 registered dermatologists in SA; 42 (16%) were based in the province of KwaZulu-Natal (KZN), serving a population of over 11 million with only 12 (4.5%) working in the public sector (Makaula et al. 2021).

Patients may turn to traditional medicine, which is more accessible in most rural areas. Moreover, they feel more comfortable and can relate to the THP. Their illnesses are explained in terms of the patient’s social-spiritual meaning systems, in contrast to Western medicine, which emphasises the disease and its pathophysiology (Satimia, Mcbride & Leppard 1998). Patients of THPs may appreciate what is perceived to be a non-judgemental, confidential and undocumented consultation. Patients of THPs may also perceive a continuation of care with one practitioner, whereas in Western biomedical contexts, the patient may experience a range of practitioners and clinical services, particularly in the public health system (Mutola et al. 2021).

There are three types of THPs identified in rural KZN*: isangoma* [diviner], *inyanga* [herbalist] and *umthandazi* [faith healer]. The THP is the custodian of the traditional African religion and fulfils roles as counsellors, mediators, spiritual protectors and educators of the African culture (Zuma et al. 2016). The THP focuses on the person in context and not on the disease (Shizha & Charema 2012). Generally, traditional healing practice constructs illness as embedded within African social, cultural and spiritual meaning systems (Naidu 2014). In traditional healing practice, the causes of ill health are attributed to social and cultural beliefs rather than the physiological or biological aetiology of the illness (Naidu 2014).

Traditional medicine plays a role in treating skin conditions. In SA, there are more than 3000 medicinal plant species, of which more than 100 are used for dermatological conditions. Plants have been shown to have antibacterial, antifungal, anti-viral, anti-inflammatory, antioxidant and analgesic properties (De Wet, Nciki & Van Vuuren 2013). Ethnobotanicals have been reported to have anti-psoriatic effects, anti-proliferative effects on squamous cell carcinoma and melanoma, and skin-lightening properties, which are open to misuse for cosmetic reasons (Dlova & Ollengo 2018).

With the promulgation of the *Traditional Health Practitioners Act* 35 of 2004, later replaced by the *Traditional Health Practitioners Act* 22 of 2007, traditional health practice received legislative recognition in SA (Moshabela & Zuma 2016; Naidu 2014). The value of THP has been recognised by the World Health Organization (WHO), advocating for the integration of THPs into the primary healthcare system (Abrams et al. 2020). Allopathic healthcare practitioners have varying views regarding the integration of THPs into the healthcare system (Mokgobi 2013). Working together may be essential for the holistic care of patients with an improvement in the outcome and compliance.

There have been studies regarding cultural practices and beliefs in the management of cancer, HIV and mental health (Akol et al. 2018; Audet, Ngobeni & Wagner 2017; Brown et al. 2018). There are no studies regarding the belief practices relating to skin conditions or rare diseases in SA.

To the authors’ knowledge, there have been no studies to date looking at THPs belief systems, perceptions and management of patients and their families with EB in SA.

## Purpose

To understand THPs belief systems, experiences, perceptions and management of EB patients and their families in the social and cultural context to improve the healthcare of EB patients, given that THPs are the first line of health consultation for many African people.

## Methods

### Research design

The study was qualitative, was conducted in two sites, used a phenomenological research design and was, therefore, orientated towards understanding the lived experience of the participants. The approach of interpretive phenomenological analysis (IPA) was used to describe and interpret the participants’ lived experiences (Smith, Flowers & Larkin 2009). Interpretive phenomenological analysis is an interpretivist approach with the goal of producing a meaningful narrative account of the participants’ perceptions and experiences rather than an objective or empirical description (Smith et al. 2009). The IPA approach was a dynamic, recursive process of gaining as accurate as possible ‘insider’s view’ through the bracketing of researcher assumptions and an engagement in the meaningful experiences of the participants (Smith et al. 2009). The phenomenological design and IPA approach matched the intention of the researchers to gain a contextual and meaningful understanding of the perceptions and experiences of the THPs. To this end, qualitative group interviews were conducted with THPs, following the life-world group interviewing approach (Dilshad & Latif 2013). The advantages of the group interview were that a rich and detailed account of the THPs experiences was produced and both the individual and shared or collective perceptions and experiences were identified. In addition to the group interview, four additional individual interviews were conducted with THPs to ascertain individual perspectives that validated or diverged from the group perceptions.

### Study setting

The first group interview, follow-up group interview and three individual interviews were held in an office at the Nelson Mandela School of Medicine in Durban, KZN, SA, a non-clinical setting familiar to the participants from previous interactions with the university. One individual interview also took place in an office at Grey’s Hospital Dermatology Department, a neutral space. Coded data were kept securely in electronic and print versions.

### Sampling, recruitment and participants

A non-probability, purposive sampling method was used. Ten THPs were recruited for the study. Inclusion criteria: a participant who is a THP registered with the Interim Council of Traditional Health Practitioners of South Africa; a representative from each THP discipline (*sangoma* [diviner], *inyanga* [herbalist] or *umthandazi* [faith healer]); gender and geographic representation in KwaZulu-Natal; and a participant who is willing to be in the study.

**Exclusion criteria:** a THP who is not registered with the Interim Council of Traditional Health Practitioners of South Africa and a THP not willing to be in the study.

They were recruited in consultation with a senior THP who had good knowledge of the individuals’ practices. The senior THP was known to members of the research team through previous studies at the university. The senior THP is registered with the Interim Council of Traditional Health Practitioners of South Africa, who invited THPs from various THP disciplines. Each THP is registered with the Interim Council of Traditional Health Practitioners of South Africa. Each THP was given an information sheet and consent form prior to commencing the study. The interviewers reviewed both documents with the participants in their preferred language, IsiZulu. Six THPs participated in group interviews, two of the six THPs then attended a follow-up interview, and four THPs participated in individual interviews. The THPs were either practising as a *sangoma* [diviner], *inyanga* [herbalist], or *umthandazi* [faith healer] or practising or had practised more than one of these disciplines (Table 1). The mean age of the THPs was 63 years, the average of 34 years of experience, with one THP quantifying her years of experience as ‘many’. The THPs were from various districts and regions of KZN and, therefore, diverse in location. The sample, therefore, represented a credible sample of ethnically Zulu and experienced traditional healers. There were no traditional healers invited who declined to participate, and none of the participants left the study.

**TABLE 1:** Sample characteristics and practices.

Respondent	Age (years)	Years of experience	Gender	Discipline(s)	Practices
Respondent 1	63	22	F	*Sangoma*	Incense; traditional medicine
Respondent 2	58	38	M	*Prophet* *Inyanga*	Incense; prayer
Respondent 3	73	Many	F	*Sangoma*	Incense; candles
Respondent 4	72	53	F	*Sangoma*	Incense; holy ash
Respondent 5	67	50	F	*Sangoma* *Inyanga*	Holy ash; incense
Respondent 6	58	22	F	*Sangoma*	Incense; prayer
Respondent 7	29	4	M	*Sangoma* *Umthandazi*	Incense, candles, prayer Oil
Respondent 8	67	50	F	*Sangoma*	Bones, prayer, holy water
Respondent 9	69	28	F	*Makhosi/Inyanga*	Prayer, incense
Respondent 10	75	47	M	*Inyanga*	Counselling, incense

F, female; M, male.

### Data collection

The interviewers were two IsiZulu-speaking postgraduate psychology students who were trained in the interview method by the primary investigator (PI) and co-supervisor before commencing the interviews. The interviewers followed a semi-structured interview schedule drafted and reviewed by the research team (Table 2). The duration of the interviews was between 60 min and 96 min. Interviews were conducted in the participants’ preferred language, which in all cases was isiZulu. The interviewers took interview notes during or immediately after each group or individual interview (debriefing meeting), transcribed each audio-recorded interview in isiZulu and then translated the interview from isiZulu to English. Following a preliminary transcript analysis, verification questions were posed in a group interview with two of the participants. In addition to the group interview, four additional individual interviews were conducted with THPs to ascertain individual perspectives that validated or diverged from the group perceptions. These interviews, therefore, functioned to enrich and triangulate the group interview material.

**TABLE 2:** Semi-structured interview schedule.

Section	Item	Interview question
Knowledge	1.	Tell me what kind of traditional practitioner you are and what consultations, treatments or rituals you perform?
	2.	In your opinion, what are the causes of disease and illness?
	3.	Tell me about your experience with this condition?
	4.	As a THP, how do you diagnose this condition?
	5.	What do you think causes this condition?
	6.	What is your cultural understanding of this condition?
Treatment	7.	How would you treat a patient with this condition?
	8.	What is the role of modern medical treatment (clinic, doctor) for this condition?
	9.	Would you advise that the patient receives medical treatment (clinic and/or doctor)? And why?
Advice	10.	What advice would you give the mother?
	11.	What advice would you give the father?
	12.	What advice would you give the family?
	13.	Should any cultural rituals be performed?
	14.	What is your advice about future pregnancies?
Prognosis	15.	What do you think is the prognosis or outcome for this baby and the family?

### Data analysis

Inductive findings were made using the systematic inductive analysis for IPA (Smith et al. 2009). Initial familiarisation with the transcripts took place with the debriefing dialogues of the PI and the interviewers. Each of the transcripts was read and re-read by the PI (A.V.C) and the co-supervisors (D.B. and C.A.) to obtain a general sense of the content, both descriptive and interpretative, and draft an initial table of themes. Annotations were made on the transcripts independently by A.V.C., D.B. and C.A., and meaningful themes were captured. The A.V.C, DB and CA devised a draft master table of themes and theme clusters and then combined and compared them independently. Links between themes were highlighted. Themes were organised in three tiers, namely basic themes, organising themes and global themes, and tabulated and structured in thematic network diagrams (Attride-Stirling 2001). During the write-up, a final integration was possible with phases of reflection and review among all three of the research team, following the IPA systematic inductive analysis, to describe and interpret the participants’ perceptions as accurately as possible (Attride-Stirling 2001). Sources of triangulation included the researcher’s reflective diary, correspondence (email or text) among the researching team and the interview information sheets with the interviewer and debriefing notes.

### Trustworthiness

Guba’s framework of trustworthiness which was further refined by Shenton was used to ensure rigour (Lincoln & Guba 1986; Shenton 2004). The elements of credibility, transferability, dependability and confirmability were used to evaluate trustworthiness. To clarify these terms from qualitative research, credibility ensures that the study measures or captures the meanings of what it intends to measure or understand. Transferability refers to the extent to which the research can be applied to various settings, in other words, the extent to which findings might be relevant in other similar settings as that of the study. Dependability refers to the extent to which reliability and replicability of the study were achieved, for example, by providing adequate information on the methods and procedures of the study. Confirmability refers to the objectivity of the study, such as through multiple sources of data or other means of triangulating findings (Shenton 2004).

Credibility was ensured through prolonged engagement and investigator triangulation using different data collection methods, including group and individual interviews, frequent debriefing sessions, member checking and iterative questioning to ensure the correct interpretation of meaning. Transferability was ensured by a thick description of the research findings which meant that the findings had a relevance to other settings, especially in sub-Saharan Africa. The use of overlapping methods and the use of and consensus among independent coders ensured dependability. Potential biases were reduced with reflexivity practices, including training of the interviewers, interviewer notes and debriefing with the PI. The interviewers were trained to elicit participation from all respondents, which reduced the risk of one or two respondents dominating the discussion. This also allowed for the divergence of views to emerge, which were further probed in the respondent validation interviews. Follow-up interviews also allowed for further engagement with participants and helped verify the emerging findings. Sources of triangulation included the researcher’s reflective diary, correspondence among the research team, interview information sheets with the interviewer and debriefing notes to ensure confirmability.

### Ethical considerations

Ethical clearance to conduct this study was obtained from the University of KwaZulu- Natal and Biomedical Research Ethics Committee of the University of KwaZulu-Natal (No. BREC/0000768/2022).

Gatekeeping permission was obtained from the health facilities. The research was conducted in accordance with the Declaration of Helsinki principles of good clinical practice and research. Each participant signed an individual informed consent after going through the information sheet with the interviewer in the isiZulu language.

## Results

Five global themes were derived from a three level thematic integration: (1) traditional health practitioner practices, (2) perceptions of THPs, (3) experiences of THPs with patients with EB, (4) diagnosis and management plans of THPs, and (5) concerns, vision, and role of THPs (Table 3). The themes were developed from the group and individual data, facilitating the triangulation of sources.

**TABLE 3:** Themes and sub-themes.

Themes	Sub-themes
1. Demographics and practices of traditional health practitioners	-
2. Perceptions of traditional health practitioners	2.1 Cultural beliefs and practices 2.2 Physical causes 2.3 Parents’ behaviour 2.4 Inherited condition
3. Traditional health practitioner’s experiences with epidermolysis bullosa	-
4. Diagnosis and management of the condition	4.1 Diagnosis 4.2 Management 4.2.1 Treatment 4.2.2 Advice given 4.3 Outcomes or prognosis
5. Concerns, vision and role of traditional health practitioners	-

### Global theme 1: Traditional health practitioners’ practices

Three male and seven female THPs were interviewed, including *sangoma, inyanga* and *umthandazi*, with an average age of 63 years and an average of 34 years of experience, with one s*angoma* quantifying her years of experience as ‘many’ (Table 1). Some THPs practices were syncretic, being both a *sangoma* and an *inyanga* or a *sangoma* and an *umthandazi*. Connecting with the ancestors was a consistent practice with all the THPs. These practices included using incense (*impepho)*, bones, candles, prayer to connect and traditional medicine (*umuthi*) or holy ash for healing.

### Global theme 2: Perceptions of traditional health practitioners

The THPs identified numerous causes of the condition, often linked to each other. Causes included:

- cultural beliefs and practices,
- physical causes,
- parents’ behaviour and
- inherited condition.

#### Sub-theme 2.1: Cultural beliefs and practices

The participants reported that a herbal remedy known as *Isihlambezo* was noted to protect the foetus during development, ensure foetal movement and ease labour. There was a shared concern that the practice of *isihlambezo* traditional practice was no longer done or was no longer done correctly by genuine THPs. This was either because people had turned away from African medicine and embraced Western medicine or, for some of the THPs, because the use of *isihlambezo* was perceived to have been blocked by health authorities and advice given at clinics:

‘*Isihlambezo* can protect the infant … banning of *isihlambezo* destroyed many things even the start of attending western cultures of attending hospitals people were told to stop taking *isihlambezo* whereas it was very helpful … people were doing wrong things like selling wrong herbs and say *isihlambezo* just to earn money … These bad herbs would result to someone being hospitalised … back in the days only old people were permitted to give out *isihlambezo* because they knew which one should be taken at which stage during pregnancy.’ (Participant 5, 67 years old, female)

The traditional healers provided explanations that linked the physical signs of the condition with an underlying spiritual malady, bad spirits or relationships, family issues or disapproving behaviours such as attempted abortions that caused disapproval or conflict among the ancestors. Completed or attempted abortion was perceived to anger the ancestors because the baby was a gift from them, and an apology needed to be offered to the ancestors:

‘… abortion in the African culture is a sin the aborted baby grows regardless even the baby who will live will suffer the sores and illness.’ (Participant 5, 67 years old, female)

A sangoma shared that these babies are born with ‘a special cloth’ and that they have a special gift from their ancestors; touching them, therefore, resulted in the presentation of the disease. They are carrying the ‘heaviness’ of ancestors and certain rituals need to be performed to alleviate this.

Relationships between kin or those from the same family clan were an area identified which could cause the condition through the ancestors’ anger:

‘There are instances where you need to perform a ritual for an angry ancestor and I also need to do the same, then there will be conflict amongst these ancestors and will result in a baby being like this.’ (Participant 1, 63 years old, female)

An incorrect ritual performed or a failure of a family to perform a ritual at the required time was believed to be causal. The THPs believed that failure to perform certain rituals when a girl entered puberty or cleansing during pregnancy with *isihlambezo* during her pregnancy would result in the baby being born with blisters. The THPs held consensual views against the perceived discontinuation or prohibition of *isihlambezo*:

‘A part of this is spirits … we believe that as traditional healers we call spirits *imindawe* or *indiki* … some things are not accepted by the ancestors … there will be something that is against the making of the baby because the ancestors do not approve.’ (Participant 2, 58 years old, male)

#### Sub-theme 2.2: Physical causes

In some of the explanations, EB was understood to be a sexually transmitted infection from the father to the mother and then to the baby. Some of the causal explanations attributed the illness to wrong foods consumed in pregnancy which could be associated with unclean blood, which results in blistering of the skin of the foetus. The associated preventative behaviour for this is related to the general care of the pregnant woman:

‘Not everything she eats is healthy and it cannot be filtered … err to a point where sometimes a baby is born with dirt, elders call this intu … his dirt is supposed to come out from her and it can be quite harmful …’ (Participant 2, 58 years old, male)

#### Sub-theme 2.3: Parent’s behaviour

A pregnant woman’s conduct and bearing were considered influential upon pregnancy outcomes, such as respect for herself, sexual morality, appropriate dress, habits and staying indoors to prevent harm to the unborn baby. Therefore, a preventable cause for the condition was perceived as appropriate conduct of the mother during the pregnancy:

‘What I understand with this illness is does not just come from nowhere all points at the mother and how she behaves and carries herself … what she eats a pregnant woman should be taken care of err and she has to love herself because this illness is result of what kind of life is the mother living.’ (Participant 6, 58 years old, female)

#### Sub-theme 2.4: Inherited condition

Some THPs noted that EB was an inherited condition:

‘The family history is important because sometimes it is inherited you will know this when you connect with ancestors … this family illness needs to be stopped traditionally.’ (Participant 5, 67 years old, female)

‘[*T*]hat children born from interracial marriages may develop this condition and believed this condition did not exist before South Africa’s democracy when interracial unions were banned.’ (Participant 10, 75 years old, male)

Despite a plethora of explanations, there was an overall feeling that EB was because of traditional African practices that had been abandoned because of Western medicine. A failure to perform rituals and cultural practices was believed to cause this condition, signifying a deeper spiritual and cultural disharmony. Causes were often tied to a preventative or curative practice such as correctly using *isihlambezo* by properly qualified THPs or performing the appropriate rituals.

### Global theme 3: Traditional health practitioner’s experiences with epidermolysis bullosa

All but one THP claimed that they had seen a patient with EB. Respondent 1 likened it to shingles and said it could be fatal. Respondent 3 reported her experience to be that traditional herbs helped a lot. Respondent 4 described an instance of the condition inherited from the father’s family and that the child was now in high school. Respondent 6 called it *umzimba omubi*, whereas respondent 5 named the condition to be *umunya* and that it was characterised as sores or bites:

‘I have seen this twice … one was big blisters that would ooze water making the baby cry … I would cook herbs and put it in a bottle and put it on top of the baby’s head than remove it and let the baby drink and bath and it would fade away … back in the days this was not called sores or blisters it was call umunya.’ (Participant 5, 67 years old, female)

Although the THPs signalled that they had an experience of the condition, the perception of the illness was quite generalised and varied among them. It was also apparent that there was no agreed-upon name for EB or one that differentiated it from other skin conditions with blisters or sores.

### Global theme 4: Diagnosis and management of the condition

The group participants consistently linked diagnosis very closely with the treatment of the condition as they perceived it. Three organising themes were identified: (1) diagnosis determines treatment, (2) management and (3) outcomes.

#### Sub-theme 4.1: Diagnosis

Consultations started with the THP enquiring about the family history of the baby, both the maternal and paternal family, primarily to determine if there was a family illness, in which case elders of the family would need to be consulted. The THPs concurred that diagnosis commenced with connecting with the ancestors to determine the cause of the condition:

‘The family history is important because sometimes it is inherited you will know this when you connect with ancestors and communicate with the parent now this family illness needs to be stopped traditionally.‘ (Participant 5, 67 years old, female)

A sangoma shared that she uses bones to connect with the ancestors and that she feels the disease that the patient is suffering from during this process.

#### Sub-theme 4.2: Management

**4.2.1 Treatment:** There was unanimous agreement that treatment was both on the ‘inside’ and the ‘outside’, and some views that Western medicine did not treat the ‘inside’:

‘To heal the baby you give something to apply and rub on the outside, but the illness is on the inside as well therefore a herb to drink for the inside, because if you only heal the outside it will eventually come back.’ (Participant 4, 72 years old, female)

Treatments were guided by the ancestors, who would reveal to the THP which rituals to perform and which herbs or combinations to use. Treatments included herbs, ash, oil, imbiza mixed with milk or holy water. Herbs were cooked or otherwise prepared; the baby would drink or bathe in the mixture. Mixtures may be rubbed on the wounds, or a herbal enema might be used in certain circumstances. One sangoma shared that he creates scarifications on the skin and applies clay to the wounds. The patient might also be required to utilise special ash for the skin lesions in several ways.

In some instances, the group discouraged concurrent use of traditional and allopathic medication, but this was not unanimous.

Cultural rituals were also performed to protect the baby, such as *imbeleko*, to cure the illness or to rectify the harm done by relationships between members of the same family clan. The required rituals were also determined by connection with the ancestors (*amadlozi*):

‘… this is common to introduce to the ancestors so the baby can be protected … the ancestors will love and protect that baby so their name will not perish.’ (Participant 5, 67 years old, female)

There were differing views regarding the need for referral to a healthcare facility. However, most did acknowledge that they would refer the patient to an allopathic healthcare facility if they could not assist the patient:

‘… if we do notice that the illness will need the western people we cannot help you we do tell you to visit the clinic and she would say I was sent by a traditional healer to come here.’ (Participant 5, 67 years old, female)

**4.2.2 Advice given:** Advice was given to the parents on care, support, treatment, prevention, not to blame each other for the baby’s illness, to comply with traditional healing advice and to do what was required to support the baby and move forward. It was also advised that parents should not assume that the baby’s illness was because of witchcraft but rather trust the traditional healer’s opinion in such a matter:

‘No matter the baby’s condition the parents must love and accept the baby … that’s what is important … what is discussed in the house of the ancestors remains confidential the only thing we deal with is love and care for the baby.’ (Participant 4, 72 years old, female)

In some instances, mothers were advised not to mix traditional and Western medicine, while others advised mothers to comply with clinic treatment.

#### Sub-theme 4.3: Outcomes and/or prognosis

The THPs noted that the condition could be cured with traditional medication and rituals. Some agreed that if they were not successful in treating a patient, they would refer the patient to another traditional healer for assistance. Some adverse outcomes were described, such as scenarios where the paternal family rejected the child or where the child was rejected or excluded by the family, the mother blamed or stigmatised including fatal outcomes.

Positive outcomes were also described, such as the belief that traditional practice could cure the individual and the family condition and that the child with EB could have a normal life:

‘They can live and be like others and the skin be repaired I know of someone who lives with this condition … there are no more scars … she has even completed matric.’ (Participant 2, 58 years old, male)

### Global theme 5: Concerns, vision and role of traditional health practitioners

There was consensus from the group that THPs played a crucial role in treating EB. Great concern was expressed that there had been a change in the behaviour of African people towards cultural beliefs and practices. The underlying feeling was that turning to Western practices to exclude traditional African practices such as the use of *isihlambezo* was the reason for illnesses and disharmony:

‘You can drink *isihlambezo* from the time of the embryo until birth of an infant. As time went by things began to change and Christianity taking over people were made to stop drinking *isihlambezo* and started clinics … then there was the start of infants being born with sores.’ (Participant 5, 67 years old, female)

There were also concerns that African traditional health knowledge was being lost, neglected or misused:

‘There is a Zulu medicine that one can lick it is ash-like that can also be applied on the sore and can also drink … this is not done anymore because now they attend hospitals.’ (Participant 5, 67 years old, female)

Some felt aggrieved that they referred patients to Western medical practitioners but that this was not reciprocated or that the health system was depriving them of their work:

‘Because we refer people to them but they will never refer people to us and not even show appreciation to just encourage and motivate us …’ (Participant 8, 67 years old, female)

The THPs shared that they were not taken seriously and viewed as uneducated by the Western health system. They also shared concern that people feared THPs and that there was a need to educate the people that they were not demon-possessed or dangerous. They also voiced concern that dishonest charlatans misrepresented traditional healing practices for financial gain. The need to protect African knowledge, skills and practices was voiced:

‘I would like to show appreciation for the opportunity given with hopes that we will continue work hand in hand. The culture is being oppressed but yet is needed for people to live and fraudsters are also trying their luck …’ (Participant 9, 69 years old, female)

The THPs appreciated the opportunity to share their views and culture during the interview. There was the perception that Western medicine relieved pain and symptoms temporarily but that traditional practices would cure EB by addressing underlying spiritual and cultural causes:

‘… it will just numb while the baby is on the traditional herb but guaranteed the traditional medication will help not doctors and clinics.’ (Participant 3, 73 years old, female)

There was general confidence in traditional health treatments and the healing abilities of THPs such that the patient would not need follow-ups as experienced in the clinic and with Western medical practitioners:

‘… they always request a follow-up on patients because once the drug is out of the system the sore will return while on our side we do not need any follow up even if you do not come back we know you will heal with what we give …’ (Participant 6, 58 years old, female)

Others took a more conciliatory position and believed that allopathic medical care was as important as traditional medicine, although this was expressed with some caveats:

‘I would advise them to go the clinic or doctors because there are things that I cannot do such as taking bloods … I am an expert when it comes to spirituality, but the clinics are expertise to what is in the body.’ (Participant 6, 58 years old, female)

Some believed that patients needed both allopathic and traditional medicine and that people should return to traditional medication, which was a gift from God:

‘… they believe that traditional meds have demons but that’s where they can only be treated … our nation needs the culture the herbs are not poisonous they are helpful Jesus gave these to help us not harm us.’ (Participant 5, 67 years old, female)

They highlighted the issue of patients’ non-disclosure of illnesses or giving a full history at Western healthcare facilities because of fear of reproach:

‘… people are even afraid to say they have problems at the clinic or come to us as traditional healers …’ (Participant 6, 58 years old, female)

The THPs shared a common vision of traditional medicine being incorporated into the healthcare system and harmoniously working together for the betterment of the patient. They appealed for traditional therapies such as *izihlambezo* to be included in the pharmacopoeia:

‘The government should make proper research to test these *izihlambezo* to see the right ones that is safe to be used.’ (Participant 6, 58 years old, female)

They also suggested that THPs could inform doctors as to the underlying cause of disease for holistic management of patients:

‘We should work together because we can see what is wrong and be able to connect with the ancestors and tell you what happened.’ (Participant 1, 63 years old, female)

Traditional health practitioners expressed the importance of reciprocal referral between themselves and the allopathic health practitioners for the holistic management of patients:

‘The department of health should work with traditional healers to fight such diseases.’ (Participant 6, 58 years old, female)

## Discussion

It is known that many African patients seek the aid of THPs before seeking allopathic healthcare (Shizha & Charema 2012). Therefore, it is imperative to understand THPs belief systems, experiences, perceptions and management of EB patients and their families in the social and cultural context to improve the healthcare of EB patients. The reasons that African patients consult with THPs as reported in the literature include accessibility, affordability, confidentiality, trustworthiness, THP being non-judgemental and their illnesses are explained according to the patient’s belief system and continuity of care (Mutola et al. 2021; Satimia et al. 1998). Patients in Africa are turning to traditional medicine for a myriad of conditions, including the treatment of HIV/AIDS, mental illness and COVID-19 infection (Bhuda & Marumo 2020; Mutola et al. 2021).

Traditional health practitioners have played an important role in HIV education and screening and improving compliance with antituberculosis and antiretroviral medication to prevent mother-to-child transmission of HIV (Peltzer 2009). They have also been noted to have been at the forefront of epidemics and pandemics in Africa such as Ebola and cholera. It was proposed that THPs be trained in the fight against the COVID-19 pandemic (Boum et al. 2021).

Acknowledging and respecting the role of traditional medicine and the THP is essential for the holistic care of patients, particularly in the psychosocial and cultural contexts of patient care.

The THPs had a plethora of knowledge and experience. Various categories of THPs used various healing practices and means of connecting with a spiritual or ancestral world, and many were syncretic in their practice.

### Perceptions of traditional health practitioners

There were multiple divergent perspectives in the room regarding the aetiology of the illness with the shared African cosmology. Interestingly, medical and cultural syncretic explanations were held by several respondents. Causes were often interlinked and converged around the omission of customary practices during pregnancy, bad spirits, ancestral influences, family relations and morality. The common cause of EB among all the THPs was the displeasure and anger of the ancestors. A lack of or inappropriate rituals that were performed was another commonality among the THPs. This was also noted by Shizha et al. that if ancestors are not appeased, and rituals are not performed, then ancestors will be angered, which may cause illness in the family (Shizha & Charema 2012).

Great emphasis was placed on the use of *isihlambezo*. This herbal mixture used by some women in KZN has many functions ranging from assistance in conceiving, preparing the cervix for delivery and protecting the newborn baby. The THPs were adamant that the discontinuation of this practice, either because of the abandonment of the African culture and following Western medicine or because of the government’s instruction, has a large role to play in this disease. Studies have shown that the use of *isihlambezo* has many effects, some being deleterious (Siveregi & Ngene 2019). The THPs advocated for the use of *isihlambezo* and suggested that further research by the Department of Health into the use of this treatment was needed.

Other causes of illness were attributed to the parent’s behaviour and moral conduct, while two THPs alluded to genetic causation. The latter is in keeping with the aetiology of the disease as defined by the allopathic healthcare system (Has et al. 2020).

Divergent views of THPs have also been noted in other fields of medicine, such as psychiatry, as to the cause of mental illnesses (Sorsdahl et al. 2010). Divergent views as to the aetiology of illness are not specific to South African THPs and have been noted in THPs in other parts of Africa (Gutema & Mengstie 2023; White 2015).

### Experience with the condition

There was no unifying term given to EB, although one THP called the condition *umzimba omubi* and another called it *umunya*. All but one THP had stated that they had seen or treated a patient with this very rare condition. It would appear that they have associated other causes of blistering conditions such as shingles (herpes infection) or sores (possible bacterial infections) with EB. There is paucity of data in South African literature. Epidermolysis is a very rare disease; it is doubtful that all but one THP had seen and treated this disease.

### Diagnosis and management of the condition

Three organising themes were identified: (1) diagnosis determines treatment, (2) management and (3) outcome and/or prognosis.

There is great importance placed on the family, the need to obtain a family history before calling on the ancestors and the need for consent from the elders before certain rituals or certain traditional medications are given. Calling on and communicating with the ancestors determines the medication that is prescribed and the rituals that need to be performed. This is analogous to allopathic medical practices that determine the diagnosis through a thorough history and appropriate investigations, which then inform treatment.

Rituals such as *imbeleko* are performed to introduce the baby by name to the ancestors and to ask for the infant’s protection (Zuma et al. 2016). Rituals are also performed to ask for forgiveness and atone for wrongdoing.

There was a consensus among all the THPs that traditional medicine aims at curing an illness internally and externally and the assumption that Western medicine can only treat the illness externally. Traditional medicine is holistic in its approach, based on social and cultural beliefs about illness and disease, focusing on the cause of illness, whereas biomedicine focuses on the symptoms (Shizha & Charema 2012). Treatment is based on the healer consulted about the illness and is tailored for that specific patient and their family (Moshabela & Zuma 2016; Shizha & Charema 2012). The patient is invited to be a part of their healing process (Moshabela & Zuma 2016). Treatments used by the THPs ranged from enemas, topical application of herbs, scarification, the use of oil, ingestion of cooked herbs and licking ash. As noted in the literature, the THPs asserted that certain conditions cannot be treated by allopathic medicine and only by traditional medicine (De Wet et al. 2013).

All the THPs were confident that they could cure EB. From a biomedical perspective, there is no documented cure for this genetic condition in the allopathic healthcare literature (Prodinger et al. 2019).

In general, concurrent use of traditional and allopathic medicine was discouraged by the THPs, the opposite of what had been reported in the literature (De Wet et al. 2013; Lampiao, Chisaka & Clements 2019). There was some divergence around the roles of allopathic medicine and traditional medicine, with some advising consecutive but not concurrent use of one or the other, contingent upon perceived outcomes of either treatment paradigm.

There were differing views as to whether THPs refer to allopathic medical facilities. The majority agreed that certain diseases were beyond their scope of practice; this was similar to the findings by Zuma et al (2016).

The advice given to the family varied among the THPs. The common theme was that love, care and acceptance were vital for the infant’s well-being. This advice was compatible with similar advice to psychosocial support given in Western medical care and treatment contexts. The THPs encouraged families not to assign blame to each other if a child is born with this disease, as opposed to what is noted in Chinese families, where blame is assigned to the mother for genetic conditions (Wu, Sun & Lee 2020). The THPs agreed that not all diseases are because of the displeasure of the ancestors and might also be because of an incidental occurrence.

There were divergent views regarding the use of traditional and allopathic healthcare practices. However, most felt that the mother should follow up with an allopathic healthcare practitioner.

### Concerns, vision and role of the traditional health practitioners

There was a great concern that people are turning away from their culture and not observing certain practices such as *isihlambezo* as they were seeking treatment at allopathic healthcare facilities.

They shared concerns that referrals to a healthcare facility were not reciprocated (Lampiao et al. 2019) and that allopathic health practitioners never acknowledge their referrals. They were seen as uneducated and thus not taken seriously, similar to findings in the literature (Akol et al. 2018; Hopa, Simbayi & Du Toit 1998; Shizha & Charema 2012). Theft of cultural intellectual property was a major concern (Mokgobi 2014). Western companies have claimed rights to traditional remedies, and local plant species are at risk of extinction (Shizha & Charema 2012).

They expressed concern that devious imposters posing as THP have given THPs a bad reputation.

Mistrust, conflict and tension have hindered collaboration between these two health systems (Akol et al. 2018; Wreford 2005). The collective vision of the THPs in this study was for collaboration and incorporation of traditional medicine into the allopathic healthcare system with a reciprocal referral system. Allopathic healthcare practitioners hold varying views in the literature. Some encourage collaboration and recognise the benefit of sharing knowledge around safe medical practices, promoting education on primary health conditions, aiding in the fight against HIV, tuberculosis and sexually transmitted infections, and learning about pharmacopoeia from THPs (Lampiao et al. 2019; Mokgobi 2014; Shizha & Charema 2012).

Other allopathic healthcare practitioners were sceptical of collaboration. African traditional healing systems are passed down verbally from generation to generation with little written records (Abrams et al. 2020; Mutola et al. 2021). Some allopathic healthcare practitioners view traditional heralthcare practices as unscientific and unsafe (Mokgobi 2013). A lack of standardisation regarding dosage, frequency, duration of treatment and side effect profile of treatment is noted (Flint & Payne 2013; Mutola et al. 2021). There have been documented cases of fatality with the consumption of traditional medicines such as *isihlambezo* (Siveregi & Ngene 2019). There is also a concern about a delay in referral to a healthcare facility that may be detrimental to a patient’s health (Nkosi & Sibiya 2021).

The vision of the THPs was that their skills be recognised, and they are incorporated into the Western healthcare system. They encouraged collaboration and reciprocal referral processes for holistic patient care.

### Recommendations

Collaboration and mutual respect between THPs and allopathic healthcare practitioners and their respective roles are essential for patients’ comprehensive care and support. Policies and programmes can be developed to improve collaboration, communications and role clarity.

Traditional healthcare practitioners could be trained to recognise skin conditions and complications that need a referral to health facilities. Traditional health practitioners are influential in the community and can play a vital role in educational programmes such as anti-skin lightening campaigns, educating the community and protecting people with albinism who have been stigmatised and accused of being witches and killed in the past (Ryan, Hirt & Willcox 2011). Research in public health and programme evaluation may help chart how to work with THPs for better community health outcomes.

Allopathic healthcare practitioners should listen to the needs of their patients and engage with the referring THP through an exchange of information and the development of a care plan that is safe and holistic for the patient. The role of the THP in the patient’s psychosocial and spiritual care should be considered in care planning.

Concerns about the use of enemas, ingestion of herbs, scarification and the topical application of herbs and concoctions should be discussed with the THP and patient or parent, especially if these are used on a patient with EB who has a loss of the protective layer of the skin epidermis and is susceptible to infections. Further research in this regard is also recommended to improve health outcomes in the community.

In-service training for both health sectors has been advocated with the introduction of traditional healing into the allopathic health curriculum (Van Rooyen et al. 2015). Pearce investigated the possibility of integrating traditional medicine into Western healthcare practice in Nigeria. Pearce found that physicians were keen to collaborate with a herbalist as herbs could be subjected to analysis for their effectiveness as opposed to other spheres of traditional medicine, such as diviners, which could not be subject to scientific objectivity or rigour (Pearce 1982). Ghana is progressive in establishing a postgraduate diploma for medical doctors (Mokgobi 2013).

The collaboration will build relationships between the healthcare and traditional care sectors, encourage rapid referrals and improve compliance and research that benefit relevant stakeholders.

### Limitations

The participants were interviewed by non-clinicians (postgraduate psychology students) outside of a treatment context. This had the advantage of diminishing potential power differentials between interviewer and interviewee, which might have reduced the interviewees from sharing openly about their differences in view from Western medical practitioners. A potential limitation of this interviewing arrangement was that had a clinician interviewed the group, there might have been the opportunity for more clinically insightful probes and further questions. The two interviewers were young Zulu women and might have felt somewhat intimidated by speaking to the culturally respected older generation. Nevertheless, the interviewers experienced the participants to be respectful and engaging.

## Conclusion

Connecting with the ancestors and doing things per the wishes of the ancestors defined all the THPs perceptions, experiences and management of EB, irrespective of their sect. They shared concerns that abandoning cultural beliefs and practices, angering the ancestors and the behaviour of the parents were causally related to EB. There were divergent views among THPs regarding the reciprocal referral to allopathic healthcare practitioners. However, many were of the opinion that traditional healthcare practices should be integrated into the allopathic healthcare practice. They shared the view that allopathic healthcare practitioners were sceptical of their practices. Certain skin care practices of THPs may be a concern to allopathic healthcare practitioners, and thus, the collaboration will result in a mutual exchange of knowledge and a change in care practices that are safe and acceptable for all. Holistic care will encourage health-seeking behaviour and improve patient compliance, and the role of THPs in the psychosocial and spiritual care of patients should be acknowledged in practice contexts, public health programmes, health system policies and medical education. Such processes will go a long way in supporting and improving the quality of life of patients affected by this challenging disease, their caregivers and their families. Mutual respect and collaboration between THPs and allopathic healthcare practitioners are thus crucial for the holistic management of patients in South Africa’s richly diverse cultural and ethnic context.
